# Three‐Channel Wavefront Shaping Using Non‐Interleaved Spin‐Multiplexed Plasmonic Metasurfaces

**DOI:** 10.1002/advs.202413138

**Published:** 2025-02-18

**Authors:** Xingling Pan, Yadong Deng, Ziru Cai, Zhiming Chen, Yingtao Ding, Ziwei Zheng, Fei Ding

**Affiliations:** ^1^ School of Integrated Circuits and Electronics Beijing Institute of Technology Beijing 100081 P. R. China; ^2^ Centre for Nano Optics University of Southern Denmark Odense DK‐5230 Denmark; ^3^ Digital Industry Research Institute Zhejiang Wanli University Ningbo 315100 P. R. China; ^4^ School of Electronic Science and Technology Eastern Institute of Technology Ningbo 315600 P. R. China

**Keywords:** beam deflector, focusing metalens, non‐interleaved plasmonic metasurfaces, spin‐multiplexed, three‐channel wavefront shaping, vortex beam generator

## Abstract

Metasurfaces have garnered significant attention for their ability to manipulate light waves with multifunctional capabilities. Integrating independent wavefront controls within a single metasurface is essential to meet the growing demand for high‐capacity, flat photonic devices. In this work, a versatile non‐interleaved plasmonic metasurface platform utilizing quarter‐wave plate meta‐atoms for independent and simultaneous phase modulation of both co‐ and cross‐polarized circularly polarized waves with subwavelength pixels, achieved by merging resonance and Pancharatnam‐Berry phases is presented. We propose and experimentally validate three proof‐of‐concept designs operating in the near‐infrared range: a beam deflector with three distinct reflection angles, a focusing metalens with three focal lengths, and a vortex beam generator with tunable topological charges. This plasmonic metasurface platform paves the way for customizable, multi‐channel functionalities, advancing the development of integrated photonic devices with enhanced versatility.

## Graphical abstract

A versatile non‐interleaved plasmonic metasurface platform has been developed using quarter‐wave plate meta‐atoms for independent and simultaneous phase modulation of both co‐ and cross‐polarized circularly polarized waves. Three proof‐of‐concept designs have been validated in the near‐infrared: a beam deflector with three distinct reflection angles, a focusing metalens with three focal lengths, and a vortex beam generator with tunable topological charges.

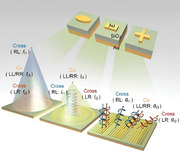

## 1 Introduction

Metasurfaces, composed of sub‐wavelength nanostructures (meta‐atoms) arranged on a two‐dimensional (2D) plane, have emerged as a prominent research focus due to their remarkable ability to manipulate light.^[^ ^1^, ^2^, ^3^, ^4^ ^]^ By precisely tuning the transmission or reflection responses of these meta‐atoms, metasurfaces can control various aspects of light, including polarization, amplitude, and phase,^[^ ^5^ ^]^ making them versatile and compact alternatives to traditional bulky optical components.^[^ ^6^, ^7^ ^]^ This versatility has led to the development of numerous applications, such as beam steering,^[^ ^8^, ^9^, ^10^ ^]^ metalenses,^[^ ^11^, ^12^ ^]^ vortex beam generation,^[^ ^13^, ^14^ ^]^ and hologram imaging.^[^ ^15^, ^16^, ^17^ ^]^

As the demand for high‐speed, large‐capacity information processing grows, the need for independent multi‐channel wavefront control with subwavelength spatial resolution has gained significant attention.^[^ ^18^, ^19^, ^20^, ^21^ ^]^ Current strategies involve either interleaved^[^ ^22^, ^23^, ^24^ ^]^ or segmented metasurfaces,^[^ ^25^, ^26^, ^27^ ^]^ which often suffer from inter‐channel crosstalk and efficiency losses between adjacent elements. While supercell pixel designs can increase the number of available channels, this comes at the cost of enlarged pixel sizes, ultimately reducing spatial resolutions.^[^ ^28^, ^29^, ^30^, ^31^ ^]^

To overcome these limitations, integrating multiple independent polarization wavefront controls into a non‐interleaved metasurface presents an attractive solution.^[^ ^32^ ^]^ The Pancharatnam‐Berry (PB) phase,^[^ ^33^, ^34^ ^]^ a spin‐dependent geometric phase associated with the rotation of meta‐atoms, has been widely employed to enable multitasking optical metasurfaces, particularly when combined with spin‐insensitive resonance or propagation phases.^[^ ^35^, ^36^, ^37^, ^38^ ^]^ However, most previous advancements have focused primarily on half‐wave plate meta‐atom designs,^[^ ^39^, ^40^, ^41^, ^42^, ^43^, ^44^, ^45^ ^]^ which controls only two cross‐polarized circularly polarized (CP) channels, overlooking the potential for co‐polarized channels.

In this work, we overcome the conventional two‐channel limitation of PB metasurfaces by utilizing plasmonic quarter‐wave plates (QWPs), enabling phase control across three CP channels with subwavelength pixels in the near‐infrared spectrum. By combining PB and resonance phases, we demonstrate independent modulation of both co‐ and cross‐polarized outputs, unlocking new possibilities for multifunctional metasurfaces. To demonstrate the feasibility of our design, we experimentally implemented a series of non‐interleaved metasurfaces with diverse functionalities, including a beam deflector with three distinct reflection angles, a focusing metalens with three different focal lengths, and a vortex beam generator with tunable topological charges. Our proposed plasmonic metasurface platform offers a flexible solution for multi‐channel wavefront control, representing a significant step toward advanced, multifunctional metasurface devices without compromising spatial resolutions.

## 2 Theory and Design

### 2.1 Principle of Three‐Channel Wavefront Shaping

For a birefringent meta‐atom orientated at an angle of *θ* with respect to the *x*‐axis, the reflective response in the CP basis can be described using the following Jones matrix (as detailed in Section S1, Supporting Information):

(1) $$R_{\mathrm{CP}}=\left(\begin{array}{cc} r_{\mathrm{LL}} & r_{\mathrm{LR}}\\ r_{\mathrm{RL}} & r_{\mathrm{RR}} \end{array}\right)=r_{\mathrm{LL}}\;\left(\begin{array}{cc} 1 & \tan\frac{\varphi_{\mathrm{yy}}-\varphi_{\mathrm{xx}}}{2}e^{i\left(-2\theta -\frac{\pi }{2}\right)}\\ \tan\frac{\varphi_{\mathrm{yy}}-\varphi_{\mathrm{xx}}}{2}e^{i\left(2\theta -\frac{\pi }{2}\right)} & 1 \end{array}\right)$$

Here, $r_{\mathrm{LL}}=|r_{\mathrm{LL}}|\;e^{i\varphi_{\mathrm{LL}}}=\left|r_{\mathrm{xx}}\right|\;\cos\frac{\varphi_{\mathrm{yy}}-\varphi_{\mathrm{xx}}}{2}e^{i(\frac{\varphi_{\mathrm{yy}}+\varphi_{\mathrm{xx}}}{2})}$, $r_{\mathrm{LR}}=\left|r_{\mathrm{LR}}\right|e^{i\varphi_{\mathrm{LR}}}$, $r_{\mathrm{RL}}=\left|r_{\mathrm{RL}}\right|e^{i\varphi_{\mathrm{RL}}}$, and $r_{\mathrm{RR}}=\left|r_{\mathrm{RR}}\right|e^{i\varphi_{\mathrm{RR}}}$ represent the CP reflection coefficients, where |*r*_xx_| is the refection amplitude, and φ_xx_ and φ_yy_ denote the reflection phases under linear polarized excitations. By introducing an appropriate rotation *θ* and controlling the phase difference between φ_xx_ and φ_yy_, it is possible to engineer three different phase patterns (φ_LL_/φ_RR_, φ_LR_, and φ_RL_), as well as distinct amplitude distributions (|*r*_LL_| = |*r*_RR_|  and |*r*_LR_| = |*r*_RL_| ). This capability enables the precise tailoring of both phase and amplitude within single meta‐atoms, allowing for the creation of highly customizable and complex optical responses from the metasurface. If the relative phase difference Δφ  = φ_yy_  − φ_xx_ equals ±90°, the meta‐atom functions as a QWP, simplifying Equation (1) to:

(2) $$R_{\mathrm{CP}}=\left|r_{\mathrm{LL}}\right|\left(\begin{array}{cc} e^{i\varphi_{\mathrm{LL}}} & e^{i\left(\varphi_{\mathrm{LL}}-2\theta -\frac{\pi }{2}\right)}\\ e^{i\left(\varphi_{\mathrm{LL}}+2\theta -\frac{\pi }{2}\right)} & e^{i\varphi_{\mathrm{LL}}} \end{array}\right)$$

This approach offers a clear pathway for precise control over three phase channels with equal intensities by carefully tuning the spin states of the input and output CP beams. Specifically, the two cross‐polarized CP components, referred to as RL (LCP to RCP) and LR (RCP to LCP) channels, exhibit phase patterns decoupled by both the PB phase (φ^PB^ =   ± 2θ) and the resonance phase ($\varphi^{\mathrm{re}}=\frac{\varphi_{\mathrm{yy}}+\varphi_{\mathrm{xx}}}{2}\;$). Meanwhile, the two co‐polarized CP components (LL and RR channels) are governed solely by the resonance phase φ^re^.

### 2.2 Meta‐Atom Design

**Figure 1:**
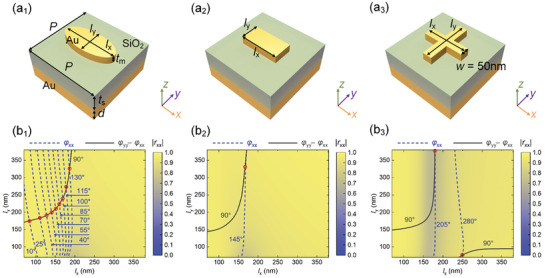
a) Schematic of the meta‐atom unit cell. b) Simulated reflection coefficient as a function of the side dimensions at the design wavelength of 850 nm for *x*‐polarized incident light: 1) ellipse; 2) brick; 3) cross‐shaped antenna. The color map shows the reflection amplitude |*r*_xx_|, while the blue dashed lines indicate contours of the reflection phase φ_xx_ with a phase step of 15°. The black solid lines highlight the meta‐atoms with a phase difference Δφ of 90°, signifying their function as QWPs.

We utilize a typical metal−insulator−metal (MIM) plasmonic resonator^[^ ^46^ ^]^ as the fundamental building block to design anisotropic QWP meta‐atoms. As illustrated in **Figure** **1**a, the plasmonic unit cell consists of a gold (Au) nanoantenna, a silicon dioxide (SiO_2_) spacer layer, and a continuous Au film. To achieve the desired phase response and maintain a high reflection amplitude, we propose five types of Au antennas, each with a thickness of *t*_m_ = 40 nm: an ellipse, a brick, and three cross‐shaped variants with different widths (*w* = 50, 70, 90 nm) (Section S2, Supporting Information). The periodicity *P* of the designed unit cells is set as 400 nm to eliminate the high‐order diffractions at the wavelength of *λ* = 850 nm. The thickness of the middle SiO_2_ spacer layer is optimized to *t*_s_ = 100 nm, ensuring high reflection efficiency and sufficient resonance phase coverage. Additionally, the thickness of the bottom Au layer is *d* = 100 nm, which is sufficiently thick to block light transmission. To determine the optimal dimensions of the meta‐atoms, 3D full‐wave simulations were performed using COMSOL Multiphysics (version 6.0). Periodic boundary conditions were applied in both *x*‐ and *y*‐directions to mimic the periodic array of unit cells, while perfectly matching layers were introduced above and below the unit cell to truncate the simulation domain and minimize boundary reflections. The relative permittivity of Au was described by the Drude model fitted to experimental data,^[^ ^47^ ^]^ whereas SiO_2_ was treated as a lossless dielectric material with a constant refractive index of 1.44. Figure 1b presents the simulated amplitudes and phase distributions of meta‐atoms with varying side dimensions (*l*_x_ and *l*_y_) at the targeted wavelength of *λ* = 850 nm under *x*‐polarized incident light, while keeping other geometrical parameters fixed. To ensure optimal efficiency, we carefully created a library of meta‐atoms, consisting of fifteen QWPs [red circles in Figure 1b] with a phase step of Δφ_xx_ =  15°. The detailed geometric parameters and corresponding serial numbers of these selected QWPs are provided in Table S1 (Supporting Information). Although the selected QWPs can't fully cover the phase range of 360°, the as‐designed metasurfaces exhibit good performance (Sections S4, S6, and S7, Supporting Information). It is worth noting that, despite our efforts to select QWPs with similar amplitude responses, minor variations in their amplitudes may still occur. While these discrepancies are minimal, they could introduce slight performance variations in the overall metasurface functionality.

## 3 Results and Discussion

### 3.1 Spin‐Multiplexed Beam Deflector

**Figure 2:**
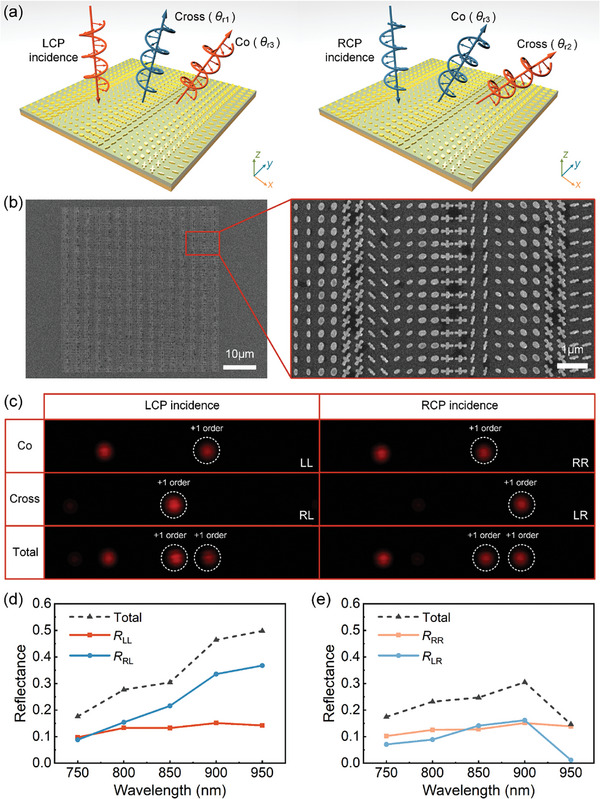
Experimental demonstration of a spin‐multiplexed three‐channel beam deflector. a) Working principle of the proposed plasmonic beam deflector under LCP and RCP incidence. Three different angles, θ_*r*1_, θ_*r*2_, and θ_*r*3_, are achieved along RL, LR, and LL/RR channels, respectively. b) SEM image of the fabricated beam deflector. c) Polarization‐resolved optical images of the diffraction spots at the wavelength of 850 nm. d,e) Experimental reflection efficiencies as a function of wavelength for d) LCP and e) RCP incident light.

Capitalizing on the constructed meta‐atom library, we first design a spin‐multiplexed beam deflector capable of generating three distinct reflection angles under LCP and RCP incident light, as illustrated in **Figure** **2**a, which relies on the modification of the tangential momentum of light within the transverse plane.^[^ ^48^ ^]^ To deflect normally incident light along the negative *z*‐direction to an angle θ_r_ in the *xz*‐plane, the metasurface imparts an appropriate phase gradient ξ along the *x*‐direction to satisfy momentum conservation, which is expressed as:

(3) $$k_{0}\;\sin\theta_{r}=\xi$$

in which $k_{0}=\frac{2\pi }{\lambda }\;$ is the wavevector in free space at the design wavelength *λ*. Considering all three channels, the following relationships can be derived from Equation (2):

(4a) $$k_{0}\;\sin\theta_{r1}=\xi_{\mathrm{RL}}=\frac{\Delta \varphi^{re}+2\Delta \theta }{P\cdot N_{\mathrm{dup}}}\;$$

(4b) $$k_{0}\;\sin\theta_{r2}=\xi_{\mathrm{LR}}=\frac{\Delta \varphi^{re}-2\Delta \theta }{P\cdot N_{\mathrm{dup}}}\;$$

(4c) $$k_{0}\;\sin\theta_{r3}=\xi_{\mathrm{LL}}=\xi_{\mathrm{RR}}=\frac{\Delta \varphi^{re}}{P\cdot N_{\mathrm{dup}}}\;$$

Here ± 2Δθ and Δφ^re^ correspond to the phase changes between neighboring meta‐atoms of different dimensions along the *x*‐direction, and *N*_dup_ =  2 represents the duplication factor used to reduce the diffraction angle of the steered light. In the design, we set ξ_RL_ =  0.1771*k*_0_ and ξ_LR_ =  0.3542*k*_0_, resulting in deflection angles of θ_r1_ ≈ 10.20° and θ_r2_ ≈ 20.74° for the cross‐polarized channels, respectively. Consequently, the third beam is directed toward $\theta_{r3}=\sin^{-1}\frac{\sin\theta_{r1}+\sin\theta_{r2}}{2}\approx 15.41^{\circ }$ as the co‐polarized channel. Based on these settings, the required resonance phases and orientations for synthesizing the complete CP channels are determined (Section S4, Supporting Information), finalizing the phase gradient metasurface for three‐channel beam deflection, as depicted in Figure 2a.

To validate the performance of the designed metasurface, we fabricated the structure using a series of well‐established nanofabrication techniques, including standard thin‐film deposition, electron beam lithography (EBL), and lift‐off methods (see the Methods section for details). Figure 2b presents a scanning electron microscopy (SEM) image of the fabricated metasurface, covering an area of ≈50 × 50 µm^2^. The SEM image reveals the fine structural details of the meta‐atoms, confirming the successful fabrication of the periodic pattern. Following fabrication, the metasurface was characterized using a custom‐built optical setup (Section S5, Supporting Information), where the sample was illuminated with CP light at normal incidence, and the reflected light was collected and analyzed to measure the polarization‐resolved reflectivities. Figure 2c shows the optical image of the diffraction spots measured at a wavelength of 850 nm, demonstrating that three distinct reflection angles were achieved, corresponding to the RL, LR, and LL/RR channels, respectively. These angles are associated with the +1‐diffraction orders, as highlighted by the white dotted circle in the figure, confirming the functionality of the metasurface for spin‐multiplexed beam deflection. To directly quantify the crosstalk in different channels, we calculate the isolation ratio in the experiment, which is defined as $\mathrm{Is}o_{i,j}=\frac{I_{i,i}}{I_{i,j}},(i,j=\mathrm{LCP},\mathrm{RCP},i\neq j)$, with *I* representing the intensity, *i* being the designed circular polarization state, and *j* indicating its orthogonal counterpart.^[^ ^49^ ^]^ The isolation ratios for the LL, RL, LR, and RR channels are 105, 282, 60, and 276, respectively, at the operating wavelength of *λ* = 850 nm, demonstrating low crosstalk and excellent performance in polarization multiplexing.

The efficiency of the metasurface was further evaluated across a broad wavelength range from 750 to 950 nm to assess its performance, as shown in Figure 2d,e. These results indicate a broadband response, showing that the metasurface operates effectively over a wide spectral range. At the target operating wavelength of *λ* = 850 nm, the total reflectivity for LCP light was measured to be 30.4%, while for RCP light, it was 24.7%. The reduced reflectivity in both cases is primarily attributed to ohmic losses in the metallic components. Despite this limitation, the metasurface demonstrates excellent control over the reflected light and successfully achieves the desired beam steering across multiple channels.

### 3.2 Spin‐Controlled Multi‐Foci Metalens

**Figure 3:**
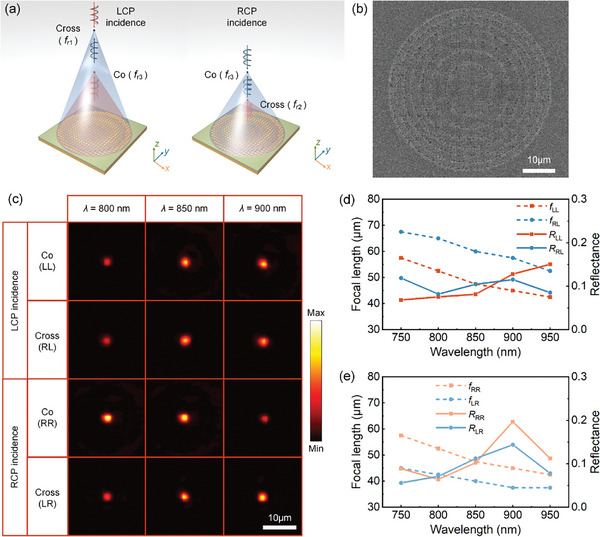
Experimental demonstration of a spin‐controlled multi‐foci metalens. a) Working principle of the proposed multi‐foci metalens under LCP and RCP incidence. Three different focal lengths, *f*_*r*1_, *f*_*r*2_, and *f*_*r*3_, are achieved along the RL, LR, and LL/RR channels, respectively. b) SEM image of the fabricated metalens. c) Intensity profiles for the different channels, measured at a distance of ≈2*f* from the focal plane of the objective at wavelengths of 800, 850, and 900 nm. d,e) Measured focal lengths and reflection efficiencies of the metalens as a function of wavelength for d) LCP and e) RCP incident light.

In addition to the beam deflector, we developed a plasmonic metalens capable of 2D focusing with three customizable focal lengths for the RL, LR, and LL/RR channels, as shown in **Figure** **3**a. This functionality requires distinct hyperbolic phase profiles for each channel, enabling the transformation of planar wavefronts into spherical wavefronts during reflection. For the RL and LR cross‐polarized channels, the phase profiles are given by the following equations:

(5a) $$\varphi_{\mathrm{RL}}\;\left(x,y\right)=360^{\circ }-\frac{360^{\circ }}{\lambda }\left(\sqrt{\left(x^{2}+y^{2}+{f_{\mathrm{RL}}}^{2}\right.}-f_{\mathrm{RL}}\right)$$

(5b) $$\varphi_{\mathrm{LR}}\;\left(x,y\right)=360^{\circ }-\frac{360^{\circ }}{\lambda }\left(\sqrt{\left(x^{2}+y^{2}+{f_{\mathrm{LR}}}^{2}\right.}-f_{\mathrm{LR}}\right)$$

where *f*_RL_ and *f*_LR_ are the desired focal lengths, and *x* and *y* represent the local coordinates of each meta‐atom. In the experiment, we fabricated a metalens sample with a 50 µm diameter, incorporating focal lengths of *f*_RL_ = 60 µm and *f*_LR_ = 40 µm for two cross‐polarization channels, whose SEM image is shown in Figure 3b. Since $\varphi_{\mathrm{LL}}\;\left(x,y\right)=\varphi_{\mathrm{RR}}\;\left(x,y\right)=\frac{\varphi_{\mathrm{RL}}\left(x,y\right)+\varphi_{\mathrm{LR}}\left(x,y\right)+\pi }{2}\;$, a third focal point is formed at $z\approx \frac{f_{\mathrm{RL}}+f_{\mathrm{LR}}}{2}$ when the difference between *f*_RL_ and *f*_LR_ is small (Section S6, Supporting Information). For characterization, we illuminated the metalens with a focused incident beam and recorded the intensity distributions when the metalens was moved away from the focal plane of the objective. Figure 3c displays the intensity distributions for different channels, measured at a distance of ≈2*f* from the focal plane of the objective at wavelengths of 800, 850, and 900 nm, showing well‐formed focal spots. Figure 3d,e show the measured focal lengths and reflection efficiencies across the working wavelength range of 750 to 950 nm under LCP and RCP excitation. As the wavelength increases, the focal length gradually decreases, consistent with the predictions of Equation (5). At the target wavelength of 850 nm, the reflected light for the RL, LR, and LL/RR channels is experimentally focused at *z*_1_ = 60 µm, *z*_2_ = 40 µm, and *z*_3_ = 47.5 µm, respectively, closely matching the designed parameters. Moreover, the fabricated metalens demonstrates a focusing efficiency of ≈10% at the wavelength of 850 nm for all channels. As the wavelength increases to 900 nm, the overall performance of the metalens improves, with slightly higher efficiency.

### 3.3 Spin‐Multiplexed Focused Vortex Beam Generator

**Figure 4:**
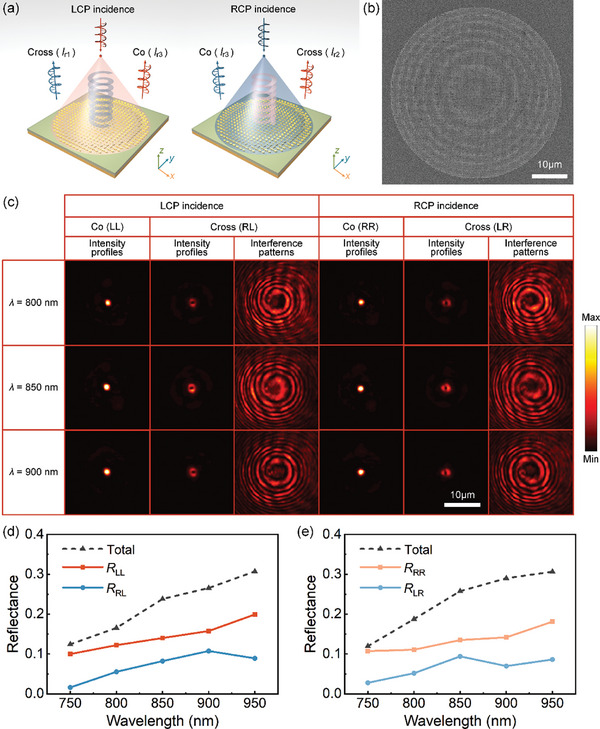
Experimental demonstration of a spin‐multiplexed focused vortex beam generator. a) Working principle of the proposed vortex beam generator under LCP and RCP incidence. Four vortex beams carrying OAM modes with topological charges, *l*_*r*1_, *l*_*r*2_, and *l*_*r*3_, are generated through the RL, LR, and LL/RR channels, respectively. b) SEM image of the focused vortex beam generator. c) Intensity profiles and interference patterns for the different channels, measured at a distance of ≈2*f* from the focal plane of the objective at wavelengths of 800, 850, and 900 nm. The interference patterns are generated by superposing the reflected light and a co‐axis reference Gaussian beam. d,e) Experimental reflection efficiencies as a function of wavelength for d) LCP and e) RCP incident light.

Building on the focusing functionality, we developed a focused vortex beam generator capable of producing orbital angular momentum (OAM) with varying topological charges across the RL, LR, and LL/RR channels, as shown in **Figure** **4**a. To generate focused vortex beams, the metasurface incorporates the phase profiles of both a lens and a q‐plate.^[^ ^13^ ^]^ Consequently, the phase distribution for the different channels imposed on the metasurface is calculated using the following formulas:

(6a) $$\varphi_{\mathrm{RL}}\;\left(x,y\right)=360^{\circ }-\frac{360^{\circ }}{\lambda }\left(\sqrt{\left(x^{2}+y^{2}+f^{2}\right.}-f\right)+l_{\mathrm{RL}}\cdot \tan^{-1}\frac{y}{x}$$

(6b) $$\varphi_{\mathrm{LR}}\;\left(x,y\right)=360^{\circ }-\frac{360^{\circ }}{\lambda }\left(\sqrt{\left(x^{2}+y^{2}+f^{2}\right.}-f\right)+l_{\mathrm{LR}}\cdot \tan^{-1}\frac{y}{x}$$

(6c) $$\begin{array}{ccc} & & \varphi_{\mathrm{LL}}\;\left(x,y\right)=\varphi_{\mathrm{RR}}\;\left(x,y\right)=450^{\circ }-\frac{360^{\circ }}{\lambda }\left(\sqrt{\left(x^{2}+y^{2}+f^{2}\right.}-f\right)\\ & & \;+\frac{l_{\mathrm{RL}}+l_{\mathrm{LR}}}{2}\cdot \tan^{-1}\frac{y}{x} \end{array}$$

where *l*_RL_ and *l*_LR_ are the topological charges for the RL and LR channels, and *f* is the focal length. For experimental validation, we fabricated a sample with a diameter of 50 µm, a focal length of 50 µm, and topological charges of ±1 for the two cross‐polarized channels (RL and LR) (Section S7, Supporting Information), resulting in zero topological charges for the co‐polarized channels (LL and RR). The SEM image of the focused vortex beam generator is shown in Figure 4b,c displays the measured intensity distributions and interference patterns for the different channels, recorded at a distance of ≈2*f* from the focal plane of the objective at wavelengths of 800, 850, and 900 nm. As expected, the RL and LR channels exhibit characteristic doughnut‐shaped intensity distributions, indicative of vortex beams with a central intensity null. Additionally, the spiral interference patterns confirm the presence of OAM with the topological charges of ±1. In contrast, the LL/RR channels display conventional Gaussian intensity profiles, reflecting their co‐polarized nature without OAM. The efficiency of the focused vortex beam generator was also measured. Figure 4d,e show the reflection efficiencies across a broad wavelength range from 750 to 950 nm for both LCP and RCP incidence. At the target wavelength of 850 nm, the total efficiency was ≈23.8% for LCP and 25.7% for RCP, demonstrating good performance in both polarization states. Furthermore, as the wavelength increases up to 950 nm, the overall efficiency improves, reflecting the wavelength‐dependent behavior of the metasurface and its robust performance across a wide spectral range.

## 4 Conclusions

In this work, we demonstrated a versatile plasmonic metasurface platform capable of spin‐multiplexed wavefront manipulation across three distinct channels. By leveraging phase control through the PB and resonance phases provided by QWP meta‐atoms, we achieved phase manipulation in the RL, LR, and LL/RR channels. This enabled the realization of optical functionalities such as beam deflection, multi‐foci metalens, and OAM‐carrying vortex beams with tunable topological charges. The experimental results from three proof‐of‐concept meta‐devices confirmed the metasurface's ability to achieve effective multi‐channel wavefront manipulation, laying a solid foundation for multifunctional integration and large‐capacity applications in advanced photonic systems. However, while the metasurface shows excellent wavefront manipulation across multiple channels at the designed wavelength, superior to other polarization‐multiplexing metasurfaces that are limited to two channels,^[^ ^41^, ^42^, ^43^, ^49^ ^]^ it is important to note that these three channels are not completely independent. The LL/RR channel is associated with the RL and LR channels due to the birefringent nature of the meta‐atoms, limiting their ability to fully decouple each channel's operation. For applications requiring complete independence among three or even four channels, future designs should integrate more advanced meta‐atom structures (i.e., supercells or double‐layer configurations)^[^ ^50^, ^51^ ^]^ or utilize appropriate elliptical polarization bases.^[^ ^52^ ^]^ In addition to polarization‐enabled multiplexing, wavelength and spatial separation can be incorporated to further increase the number of available channels,^[^ ^49^, ^53^, ^54^ ^]^ thereby unlocking the greater potential for complex optical functionalities and advanced photonic devices. With great potential, our platform remains highly functional, offering additional spin‐controlled channels that significantly expand the possibilities for wavefront engineering.

## 5 Experimental Section

### Fabrication

The standard thin‐film deposition, electron‐beam lithography, and lift‐off techniques to fabricate the metasurfaces was used. Initially, a silicon substrate experienced sequential thermal evaporation of layers: 3 nm of Ti, 100 nm of Au, and an additional 1 nm of Ti. Subsequently, an RF‐sputtered deposition resulted in the formation of a 100 nm SiO_2_ spacer layer. Following this, a 100 nm PMMA layer (2% in anisole, Micro Chem) was spin‐coated onto the SiO_2_ layer, baked at 180 °C for 2 min, and then subjected to pattern definition through exposure at an acceleration voltage of 30 keV. After exposure, the wafer was developed in a solution containing methyl isobutyl ketone (MIBK) and isopropyl alcohol (IPA) at a ratio of MIBK to IPA of 1:3 for 35 s, followed by immersion in an IPA bath for 60 s. Post‐development, a 1 nm Ti adhesion layer and a 40 nm Au layer were sequentially deposited via thermal evaporation. Ultimately, top Au nanobricks were obtained by a lift‐off process.

## Conflict of Interest

The authors declare no conflict of interest.

## Supporting information



Supporting Information

## Data Availability

The data that support the findings of this study are available from the corresponding author upon reasonable request.
