# Supplementary material for: Three‐Channel Wavefront Shaping Using Non‐Interleaved Spin‐Multiplexed Plasmonic Metasurfaces
Source: Adv Sci (Weinh). 2025 Feb 18;12(17):2413138. doi: 10.1002/advs.202413138 (PMC12061329; doi:10.1002/advs.202413138)
Supplement: Supplementary file 1 — Supporting Information [file ADVS-12-2413138-s001.pdf]

ADVANCED SCIENCE
Open Access

Supporting Information

for *Adv. Sci.*, DOI 10.1002/advs.202413138

Three-Channel Wavefront Shaping Using Non-Interleaved Spin-Multiplexed Plasmonic Metasurfaces

Xingling Pan, Yadong Deng, Ziru Cai, Zhiming Chen, Yingtao Ding, Ziwei Zheng and Fei Ding**

Supporting Information

Three-Channel Wavefront Shaping Using Non-Interleaved Spin-Multiplexed Plasmonic Metasurfaces

Xingling Pan, Yadong Deng, Ziru Cai, Zhiming Chen, Yingtao Ding, Ziwei Zheng, and Fei Ding**

Section S1. Derivation of the Jones matrix for an QWP meta-atom

For a general planar, reciprocal 2D meta-atom in a reflection scheme, we can use the Jones matrix to describe its response in the CP basis:

$$R_{\mathrm{CP}} = \begin{pmatrix} r_{\mathrm{LL}} & r_{\mathrm{LR}} \\ r_{\mathrm{RL}} & r_{\mathrm{RR}} \end{pmatrix} = \Lambda^{-1} M^{-1}(\theta) \begin{pmatrix} r_{\mathrm{xx}} & r_{\mathrm{xy}} \\ r_{\mathrm{yx}} & r_{\mathrm{yy}} \end{pmatrix} M(\theta) \Lambda \tag{S1}$$

Here, $r_{\mathrm{LL}} = |r_{\mathrm{LL}}| e^{i\varphi_{\mathrm{LL}}}$, $r_{\mathrm{LR}} = |r_{\mathrm{LR}}| e^{i\varphi_{\mathrm{RL}}}$, $r_{\mathrm{RL}} = |r_{\mathrm{RL}}| e^{i\varphi_{\mathrm{LR}}}$, and $r_{\mathrm{RR}} = |r_{\mathrm{RR}}| e^{i\varphi_{\mathrm{RR}}}$ are the CP reflection coefficients, while $r_{\mathrm{xx}} = |r_{\mathrm{xx}}| e^{i\varphi_{\mathrm{xx}}}$, $r_{\mathrm{xy}} = |r_{\mathrm{xy}}| e^{i\varphi_{\mathrm{xy}}}$, $r_{\mathrm{yx}} = |r_{\mathrm{yx}}| e^{i\varphi_{\mathrm{yx}}}$, and $r_{\mathrm{yy}} = |r_{\mathrm{yy}}| e^{i\varphi_{\mathrm{yy}}}$ represent the LP reflection coefficients of the meta-atom without any rotation (θ = 0), where the first and second subscripts denote the output and input waves, respectively. $M(\theta) = \begin{pmatrix} \cos\theta & \sin\theta \\ -\sin\theta & \cos\theta \end{pmatrix}$ is the rotation matrix and $\Lambda = \frac{1}{\sqrt{2}} \begin{pmatrix} 1 & 1 \\ i & -i \end{pmatrix}$ is the transformation matrix. Therefore, the four CP reflection coefficients are expressed as:

$$r_{\mathrm{LL}} = \frac{1}{2}\left[(r_{\mathrm{xx}} + r_{\mathrm{yy}}) + i(r_{\mathrm{xy}} - r_{\mathrm{yx}})\right] \tag{S2a}$$

$$r_{\mathrm{LR}} = \frac{1}{2}\left[(r_{\mathrm{xx}} - r_{\mathrm{yy}}) - i(r_{\mathrm{xy}} + r_{\mathrm{yx}})\right] e^{-i2\theta} \tag{S2b}$$

$$r_{\mathrm{RL}} = \frac{1}{2}\left[(r_{\mathrm{xx}} - r_{\mathrm{yy}}) + i(r_{\mathrm{xy}} + r_{\mathrm{yx}})\right] e^{i2\theta} \tag{S2c}$$

$$r_{\mathrm{RR}} = \frac{1}{2}\left[(r_{\mathrm{xx}} + r_{\mathrm{yy}}) - i(r_{\mathrm{xy}} - r_{\mathrm{yx}})\right] \tag{S2d}$$

Considering a birefringent meta-atom with its principal axes along the *x*- and *y*-directions ($r_{\mathrm{xy}} = r_{\mathrm{yx}} = 0$) and assuming equal reflection amplitudes ($|r_{\mathrm{xx}}| \approx |r_{\mathrm{yy}}|$), Equation (S2) simplifies to:

$$r_{\mathrm{LL}} = |r_{\mathrm{xx}}| \cos\frac{\varphi_{\mathrm{yy}} - \varphi_{\mathrm{xx}}}{2} e^{i\left(\frac{\varphi_{\mathrm{yy}} + \varphi_{\mathrm{xx}}}{2}\right)} \tag{S3a}$$

$$r_{\mathrm{LR}} = |r_{\mathrm{xx}}| \sin\frac{\varphi_{\mathrm{yy}} - \varphi_{\mathrm{xx}}}{2} e^{i\left(\frac{\varphi_{\mathrm{yy}} + \varphi_{\mathrm{xx}}}{2} - \frac{\pi}{2} - 2\theta\right)} \tag{S3b}$$

$$r_{\mathrm{RL}} = |r_{\mathrm{xx}}| \sin\frac{\varphi_{\mathrm{yy}} - \varphi_{\mathrm{xx}}}{2} e^{i\left(\frac{\varphi_{\mathrm{yy}} + \varphi_{\mathrm{xx}}}{2} - \frac{\pi}{2} + 2\theta\right)} \tag{S3c}$$

$$r_{\mathrm{RR}} = |r_{\mathrm{xx}}| \cos\frac{\varphi_{\mathrm{yy}} - \varphi_{\mathrm{xx}}}{2} e^{i(\frac{\varphi_{\mathrm{yy}}+\varphi_{\mathrm{xx}}}{2})} = r_{\mathrm{LL}} \tag{S3d}$$

Equation (S3) indicates that three different phase patterns ($\varphi_{\mathrm{LL}}/\varphi_{\mathrm{RR}}$, φ_{LR}, and φ_{RL}), as well as distinct amplitude distributions ($|r_{\mathrm{LL}}| = |r_{\mathrm{RR}}|$ and $|r_{\mathrm{LR}}| = |r_{\mathrm{RL}}|$), can be engineered by adjusting the rotation angle θ and the phase difference between φ_{xx} and φ_{yy}. Substituting Equation (S3) into Equation (S1), we obtain:

$$R_{\mathrm{CP}} = |r_{\mathrm{xx}}| \cos\frac{\varphi_{\mathrm{yy}} - \varphi_{\mathrm{xx}}}{2} e^{i\left(\frac{\varphi_{\mathrm{yy}}+\varphi_{\mathrm{xx}}}{2}\right)} \begin{pmatrix} 1 & \tan\frac{\varphi_{\mathrm{yy}} - \varphi_{\mathrm{xx}}}{2} e^{i\left(-\frac{\pi}{2} - 2\theta\right)} \\ \tan\frac{\varphi_{\mathrm{yy}} - \varphi_{\mathrm{xx}}}{2} e^{i\left(-\frac{\pi}{2} + 2\theta\right)} & 1 \end{pmatrix} \tag{S4}$$

If the relative phase difference $\Delta\varphi = \varphi_{\mathrm{yy}} - \varphi_{\mathrm{xx}}$ equals ±90°, the anisotropic meta-atom functions as a QWP, simplifying Equation (S4) to:

$$R_{\mathrm{CP}} = |r_{\mathrm{LL}}| \begin{pmatrix} \mathrm{e}^{i\varphi_{\mathrm{LL}}} & \mathrm{e}^{i(\varphi_{\mathrm{LL}} - 2\theta - \frac{\pi}{2})} \\ \mathrm{e}^{i(\varphi_{\mathrm{LL}} + 2\theta - \frac{\pi}{2})} & \mathrm{e}^{i\varphi_{\mathrm{LL}}} \end{pmatrix} \tag{S5}$$

Therefore, the co-polarized channels (LL/RR) are interdependent with the cross-polarized channels (RL/LR) as their phases are associated (i.e., $\varphi_{\mathrm{LL}} = \varphi_{\mathrm{RR}} = \frac{\varphi_{\mathrm{LR}}+\varphi_{\mathrm{RL}}+\pi}{2}$).

Section S2. Cross-shaped meta-atoms with widths of 90 and 70nm

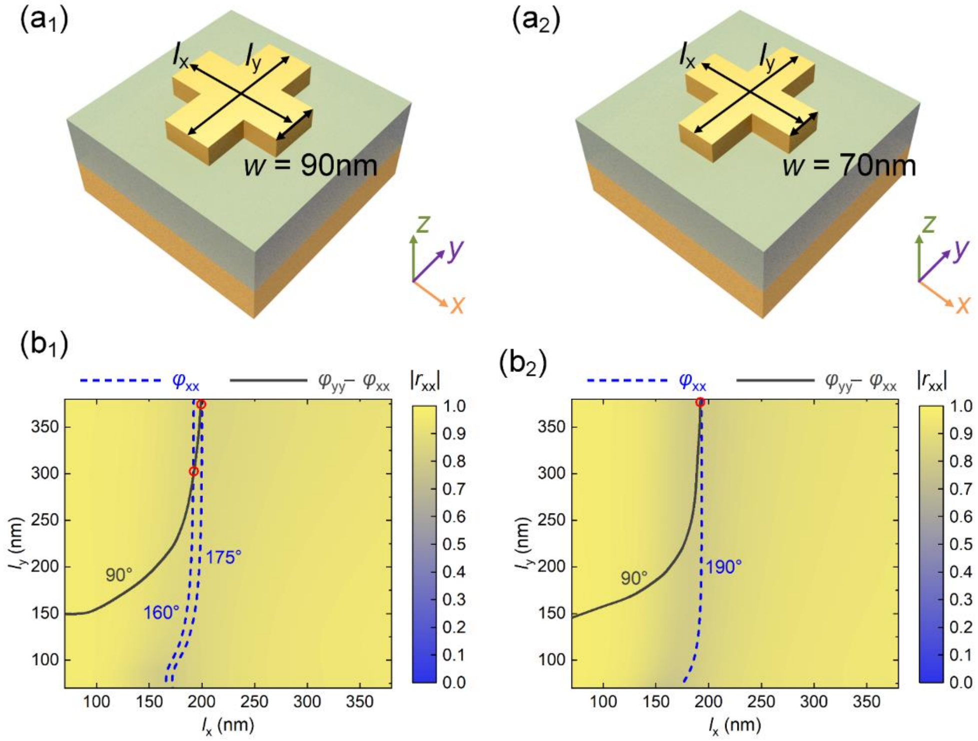

Figure S1. a) Schematic of the cross-shaped meta-atom unit cell. b) Simulated reflection coefficient as a function of the dimensions at the design wavelength of 850 nm for *x*-polarization: cross-shaped antenna with widths of 1) 90 and 2) 70 nm. The color map shows the reflection amplitude $|r_{xx}|$, while the blue dashed lines indicate contours of the reflection phase φ_{xx} with a phase step of 15°. The black solid lines highlight the meta-atoms with a phase difference $\Delta\varphi$ of 90°, indicating their function as QWPs.

Section S3. Dimensions and reflection coefficients of the selected 15 QWP meta-atoms

Table S1. Dimensions and reflection coefficients of the selected 15 meta-atoms.

**No. of the meta-atom**	**shape**	l_x**/nm**	l_y**/nm**	φ_{xx}**/deg**	$\Delta\varphi$**/deg**	$\lvert r_{xx} \rvert$
1	ellipse	84	175	10.1	89.8	0.97
2	ellipse	111	184	25.3	89.6	0.96
3	ellipse	128	192	40.4	89.5	0.95
4	ellipse	140	200	54.5	89.1	0.94
5	ellipse	151	212	70.3	90.8	0.94
6	ellipse	160	224	84.7	90.2	0.93
7	ellipse	169	243	100.2	90.8	0.93
8	ellipse	178	270	115.6	89.8	0.93
9	ellipse	187	325	130.9	89.3	0.94
10	brick	165	325	141.9	90.8	0.94
11	cross-shaped (w = 90 nm)	192	300	160.4	90.5	0.86
12	cross-shaped (w = 90 nm)	200	385	175.3	89.7	0.85
13	cross-shaped (w = 70 nm)	193	392	190.0	89.5	0.80
14	cross-shaped (w = 50 nm)	180	395	206.0	88.4	0.71
15	cross-shaped (w = 50 nm)	250	77	280.3	90.0	0.90

Section S4. Design of a spin-multiplexed three-channel beam deflector

Figure S2a and Figure S2b present the required resonance phases and rotation angles for synthesizing complete CP channels, utilizing twelve atoms with varying shapes, dimensions, and orientations. Figure S2c shows part of the metasurface layout, where the meta-atoms are duplicated twice along the *x*-axis to reduce the diffraction angle and arranged periodically along the *y*-axis.

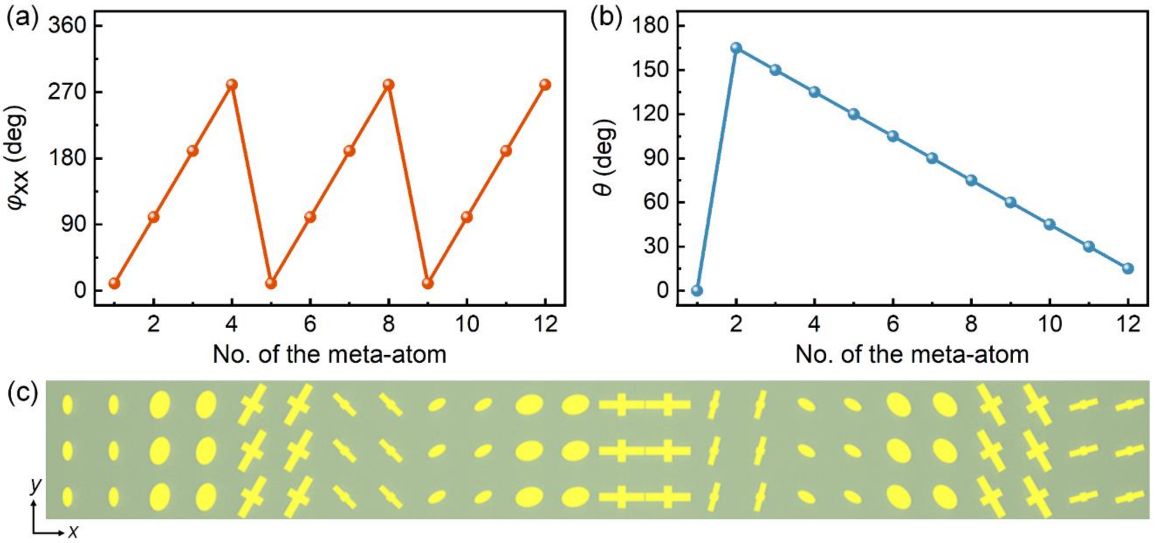

Figure S2. a) Resonance phase φ_{xx} and b) rotation angle θ of the designed spin-multiplexed three-channel beam deflector. c) Layout of the spin-multiplexed three-channel beam deflector.

To validate the performance, we conduct 3D simulations using the finite-difference time-domain (FDTD) method with a commercial software package (Lumerical), where near-field distributions are projected into the far field. Figure S3 plots far-field intensity distributions and cross-section intensity profiles along different angles at the operating wavelength of 850 nm. As anticipated, three beams with different deflection angles are found in RL, LR, and LL/RR channels, directed toward $\theta_{r1} = 10.25°$, $\theta_{r2} = 20.69°$, and $\theta_{r3} = 15.56°$, respectively. These values closely align with the theoretically calculated values of $\theta_{r1} = 10.21°$, $\theta_{r2} = 20.74°$, and $\theta_{r3} = 15.4°$. The RL and LL/RR channels exhibit similar intensities, whereas the LR channel shows a comparatively lower intensity, possibly due to the inconsistent amplitudes of the selected meta-atoms. It's worth noting that there is no phase gradient along the *y*-direction, forming a line intensity distribution along the *y*-axis in the far field (Figure S3a,c). In addition, the absolute reflection efficiency, defined as the intensity ratio between the reflected and incident light, exceeds 80.6% and 80.2% under LCP and RCP excitation, respectively.

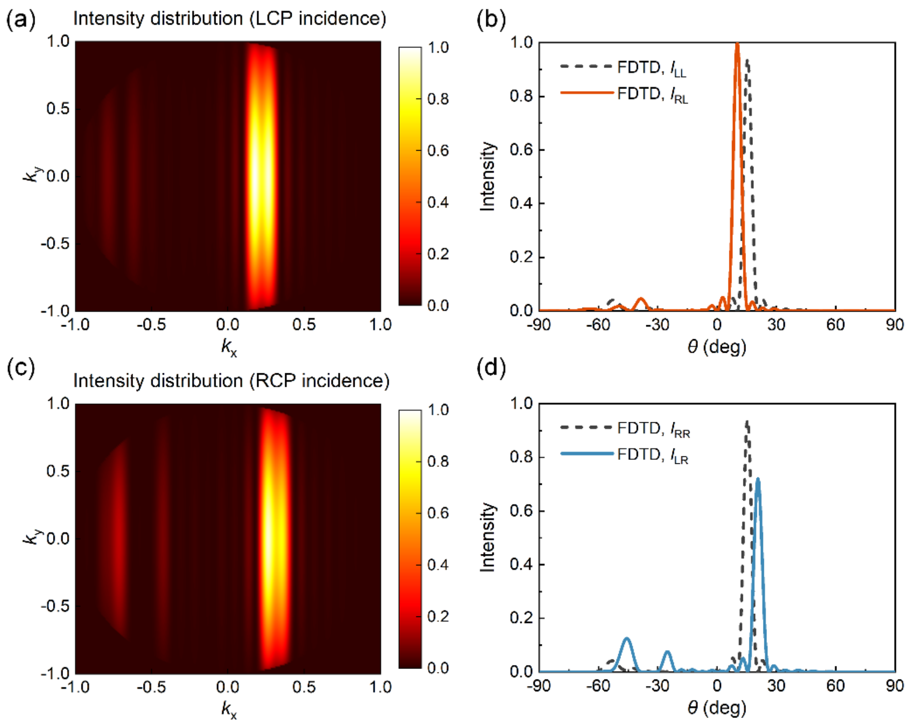

Figure S3. a,c) Simulated far-field intensity distributions for a) LCP and c) RCP incident light. b,d) The normalized cross-section intensity profiles for b) LCP and d) RCP incident light. All intensity distributions have been normalized.

Section S5. The custom-built optical setup

Figure S4 shows the schematic of the experimental setup. A collimated fiber-coupled continuous-wave Ti:sapphire laser (Spectra-Physics 3900 S, wavelength from 700 to 1000 nm) first passes through a linear polarizer (LP1, LPNIR050-MP2, Thorlabs) and an attenuator (NE01B, Thorlabs) to control the light intensity. The light is then reflected by a silver mirror (PF10-03-P01, Thorlabs) and directed through another linear polarizer (LP2, LPNIR050-MP2, Thorlabs) and a quarter-wave plate (QWP1, AQWP10M-980, Thorlabs) on a motorized rotation stage (PRM1Z8, Thorlabs). It then passes through two beam splitters (BS1 and BS2, CCM1-BS014/M, Thorlabs) before being slightly focused onto the sample by a long working distance objective (M Plan Apo 20×/0.42NA, Mitutoyo). The combination of LP2 and QWP1 generates left circularly polarized (LCP) and right circularly polarized (RCP) light in a controlled manner. Two beam splitters adjust the beam direction and compensate for polarization-dependent phase shifts that would occur if a single beam splitter was used. The reflected signal light, collected by the same objective, passes through BS2 and a tube lens (TTL200-S8, f = 200 mm, Thorlabs), creating the initial direct image plane. An iris (SM1D12SZ, Thorlabs) is positioned at this plane to filter out background signals. The filtered initial real image undergoes further transformation by a relay lens (RL, AC254-200-B-ML, f = 200 mm, Thorlabs) to produce a Fourier image, which is captured by a CMOS camera (DCC1545M, Thorlabs). To construct the co- and cross-polarized channels, we employ a quarter-wave plate (QWP2, AQWP10M-980, Thorlabs) and a linear polarizer (LP3, LPNIR050-MP2, Thorlabs). Additionally, a flip lens (FL, AC254-100-B-ML, f = 100 mm, Thorlabs) is positioned between the relay lens and the CCD to alternate between real images (for metalens characterization, z = 2f) and Fourier images (for beam steering characterization).

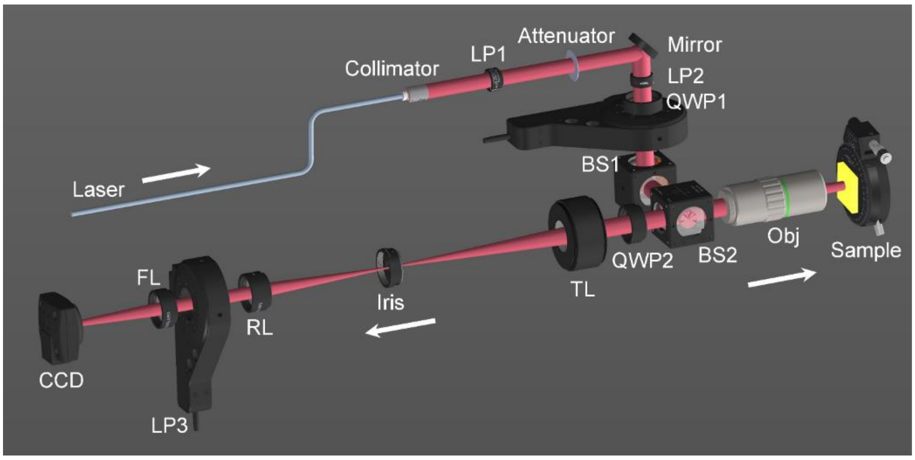

Figure S4. Schematic of the experimental setup for characterizing metasurfaces

Section S6. Design of a spin-controlled multi-foci metalens

We designed a spin-controlled multi-focal metalens with a 50 μm diameter and focal lengths of f_{RL} = 60 μm and f_{LR} = 40 μm for two cross-polarization channels at the target wavelength of 850 nm. By combining Equation (2) and Equation (5), we computed the required resonance phase distributions and determined the specific orientations of the meta-atoms at each position across the metalens surface, as shown in Figure S5a,b. Figure S5c presents the theoretical phase profiles for the RL, LR, and LL/RR channels along the x-axis for y = 0, where $\varphi_{\mathrm{LL}}(x,y) = \varphi_{\mathrm{RR}}(x,y) = \frac{\varphi_{\mathrm{RL}}(x,y)+\varphi_{\mathrm{LR}}(x,y)+\pi}{2}$. The calculated phase profile for a lens with a focal length of $z = \frac{f_{\mathrm{RL}}+f_{\mathrm{LR}}}{2}$ is also shown, which closely aligns with the theoretical phase.

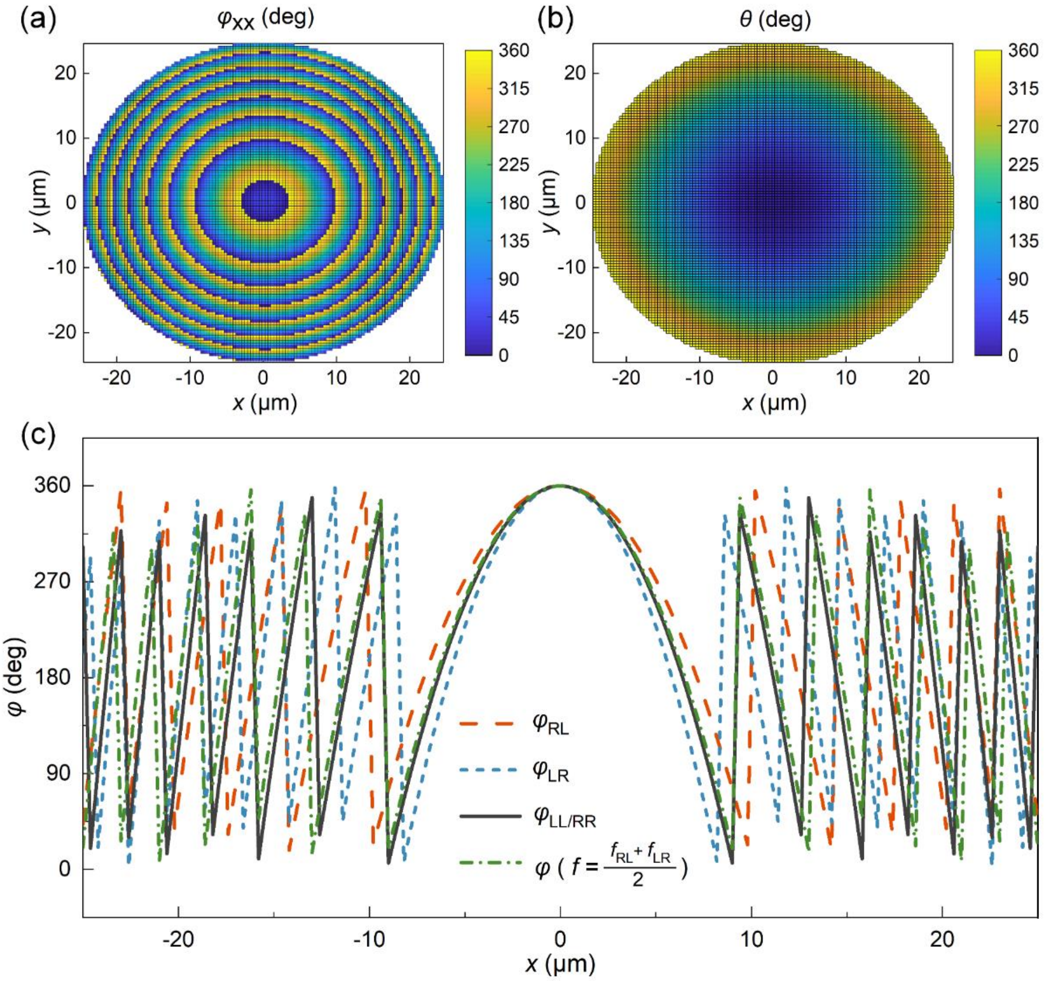

Figure S5. a) Resonance phase φ_{xx} and b) rotation angle θ of the designed spin-controlled multi-foci metalens. c) Phase profiles for RL, LR, and LL/RR channels, alongside the calculated phase profile of a lens with a focal length of $\frac{f_{\mathrm{RL}}+f_{\mathrm{LR}}}{2}$, shown along the x-axis for y = 0.

We conducted 3D FDTD simulations to numerically investigate the performance of the designed multi-foci metalens with a reduced size. The metalens has a diameter of 10 μm and is

configured with focal lengths of 8 μm and 12 μm for the two cross-polarization channels. These parameters are consistent with the numerical aperture of the experimental sample. Figure S6 shows the decomposed intensity distributions of the reflected light in the CP basis in the *x*–*z* plane and the intensity profiles along the *z*-axis at $x = y = 0$ at the operating wavelength of 850 nm. The results demonstrate that the reflected light is well focused at $z_1 = 7.2$ μm, $z_2 = 11.1$ μm, and $z_3 = 8.9$ μm for the RL, LR, and LL/RR channels, respectively, aligning well with the designed parameters.

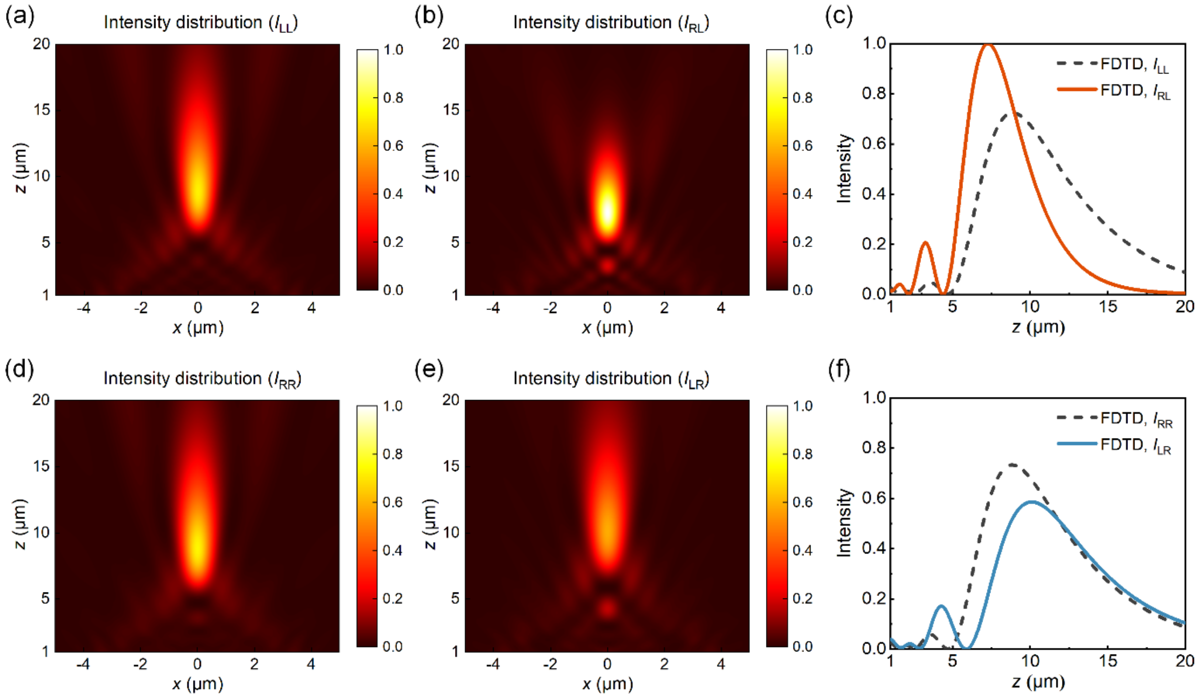

Figure S6. a,b,d,e) Simulated intensity distributions of the a,e) LCP and b,d) RCP components for a,b) LCP and d,e) RCP incident light. c,f) The normalized cross-section intensity profiles along the *z*-axis at $x = y = 0$ in the CP basis for c) LCP and f) RCP incident light. All intensity distributions have been normalized.

Section S7. Design of a spin-multiplexed focused vortex beam generator

We developed a spin-multiplexed focused vortex beam generator with a diameter of 50 μm, a focal length of 50 μm, and topological charges of $l = \pm 1$ for two cross-polarization channels, operating at a wavelength of 850 nm. Following the approach outlined in Section S6, we use Equation (2) and Equation (6) to determine the required resonance phase distributions and meta-atom orientations at each position, as depicted in Figure S7.

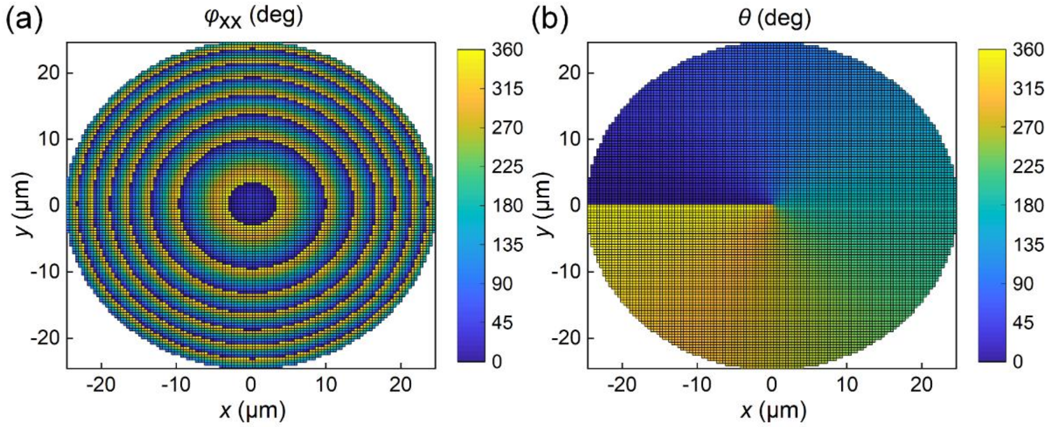

Figure S7. a) Resonance phases φ_{xx} and b) rotation angle θ of designed spin-multiplexed focused vortex beam generator.

The simulated vortex beam generator with a scaled-down dimension has a diameter of 10 μm, a focal length of $f = 10$ μm, and topological charges of $l = \pm 1$ for two cross-polarization channels. These values align with the numerical aperture of the experimental sample. The left column of Figure S8 shows the decomposed intensity distributions of the reflected light in the CP basis in the x–z plane at the operating wavelength of 850 nm. The reflected light is well focused at 8.9 μm and 9.3 μm in the RL/LR and LL/RR channels, respectively. The middle and right columns of Figure S8 present the simulated intensity profiles and phase distributions of two CP components in the focal plane. Under LCP and RCP excitations, the doughnut-shaped intensity distributions (Figure S8e,h) confirm the existence of vortex beams, corresponding to the RL/LR channels. The spiral phase distributions in intensity regions reveal 2π phase variation along the azimuthal direction with opposite signs (Figure S8f,i), identifying the topological charges of $l = \pm 1$ for the cross-polarized OAMs. On the other hand, the LL/RR channels show Gaussian intensity distribution (Figure S8b,k), with constant phases (Figure S8c,l). Additionally, under LCP or RCP incidence, the two orthogonal CP components of the reflected light account for 48.7% and 51.3% of the total energy, respectively, indicating that the energies carried by the two components are essentially equal.

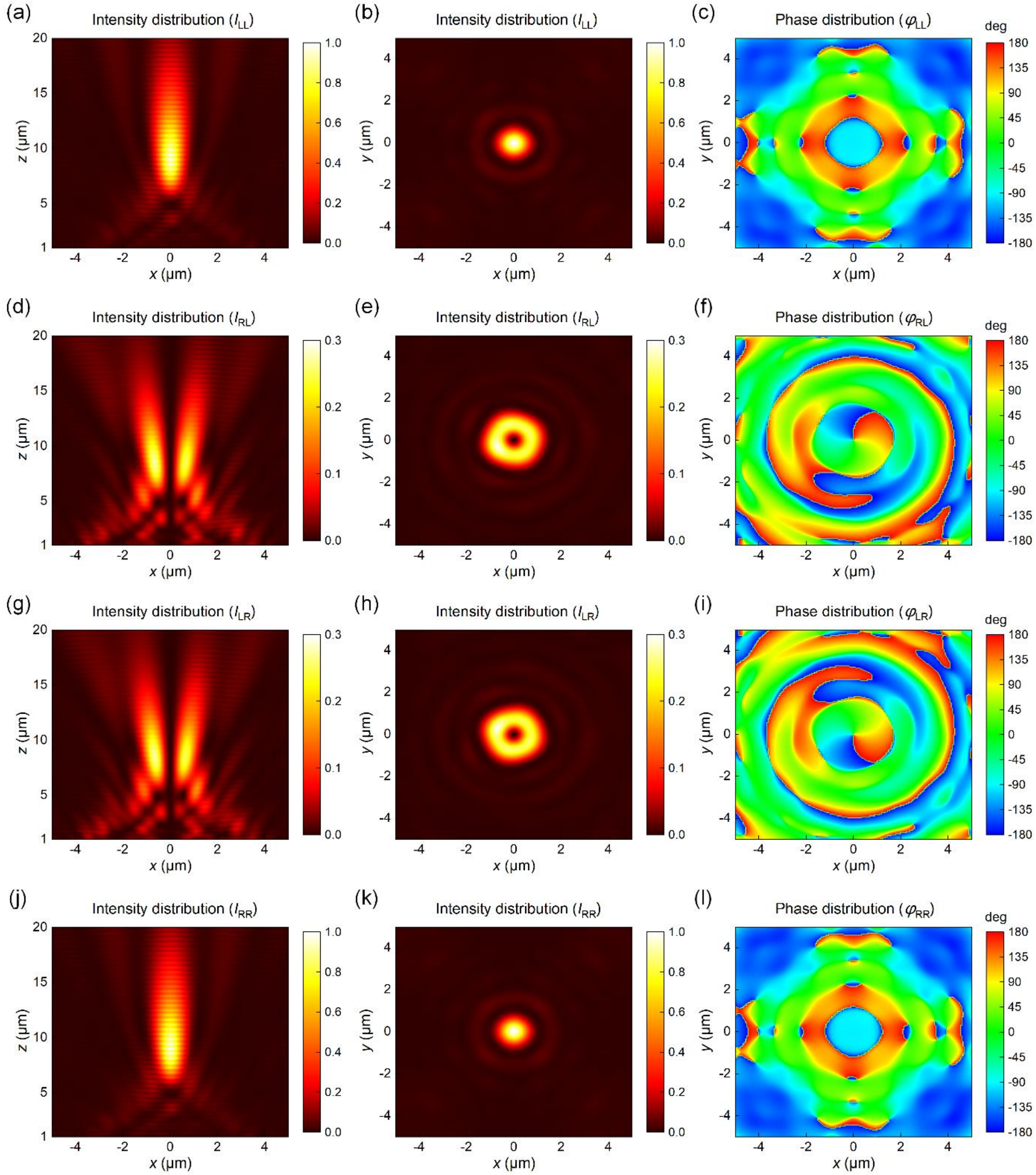

Figure S8. Simulated a,b,d,e,g,h,j,k) intensity and c,f,i,l) phase distributions of the designed spin-multiplexed focused vortex beam generator with a small size for a-f) LCP and g-l) RCP incident light. All intensity distributions have been normalized.

To quantitatively verify the OAM emission, we projected decomposed cross- and co-polarized CP components into the OAM basis, encoding spiral phases corresponding to specific topological charges. Subsequently, we determined the OAM mode purity, defined as the relative central intensity of the beam after demodulation by the spiral phase with an opposite topological charge[1]. As depicted in Fig. S9, the mode purities of RL and LR channels with

topological charges of $l_{RL} = 1$ and $l_{LR} = -1$ are determined to be approximately 0.833. For the LL/RR channel carrying no OAMs, the mode purity values are around 0.925.

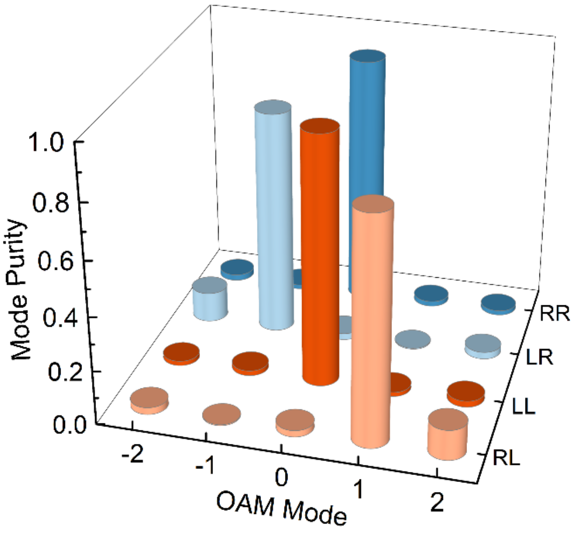

Figure S9. Calculated mode purities of two CP components for LCP and RCP incidences.

References

[1] J. Zhang, C. Sun, B. Xiong, J. Wang, Z. Hao, L. Wang, Y. Han, H. Li, Y. Luo, Y. Xiao, *Nat. Commun.* **2018**, 9, 2652.
